# Comparative Neuropsychiatric Outcomes of JAK Inhibitors, Dupilumab, and Conventional Immunosuppressants in Atopic Dermatitis: A Real-World Cohort Study

**DOI:** 10.3390/biomedicines14071482

**Published:** 2026-06-30

**Authors:** Chang-Ching Wei, Tsung-Ju Li, Hao-Yun Chen, Jing-Yang Huang, Ting-Yuan Lin, Jiu-Yao Wang, Wen-Ling Liao, James Cheng-Chung Wei

**Affiliations:** 1 Division of Allergy, Immunology and Rheumatology, Department of Pediatrics, China Medical University Children’s Hospital, Taichung 404327, Taiwan; weilonger@gmail.com (C.-C.W.); 028537@tool.caaumed.org.tw (T.-J.L.);; 2 School of Medicine, China Medical University, Taichung 404333, Taiwan; 3 Institute of Medicine, Chung Shan Medical University, Taichung 40201, Taiwant.ylin0705@gmail.com (T.-Y.L.); 4 Center for Health Data Science, Chung Shan Medical University Hospital, Taichung 40201, Taiwan; 5 Center of Allergy, Immunology and Microbiome (AIM), Department of Allergy and Immunology, China Medical University Children’s Hospital, Taichung 404327, Taiwan; 6 Graduate Institute of Integrated Medicine, College of Chinese Medicine, China Medical University, Taichung 40402, Taiwan; 7 Center for Personalized Medicine, Department of Medical Research, China Medical University Hospital, Taichung 40447, Taiwan; 8 Department of Allergy, Immunology and Rheumatology, Chung Shan Medical University Hospital, Taichung 40201, Taiwan

**Keywords:** atopic dermatitis, depression, anxiety, JAK inhibitors, dupilumab, mental health

## Abstract

**Background:** Atopic dermatitis (AD) is associated with neuropsychiatric comorbidity, yet no study has directly compared neuropsychiatric outcomes across all three major systemic treatment classes. This study examined neuropsychiatric outcomes among patients with AD receiving Janus kinase (JAK) inhibitors, dupilumab, or conventional immunosuppressants. **Methods:** This retrospective cohort study utilized the TriNetX global health research network. Three propensity score-matched cohorts were constructed based on demographics, comorbidities, and concomitant medications. The primary outcome was incident neuropsychiatric disorder within 24 months. **Results:** After matching, cohorts included 608 (JAK inhibitors vs. dupilumab), 502 (JAK inhibitors vs. conventional), and 2322 patients (dupilumab vs. conventional). In the largest cohort, conventional immunosuppressants were associated with a significantly higher incidence of neuropsychiatric disorders compared with dupilumab (hazard ratio [HR] 1.26, 95% confidence interval [CI] 1.00–1.59; *p* = 0.042). A similar directional trend was observed for conventional immunosuppressants compared with JAK inhibitors (HR 1.97, 95% CI 1.13–3.42; *p* = 0.015). The comparison between JAK inhibitors and dupilumab did not reach statistical significance (HR 0.62, 95% CI 0.37–1.03; *p* = 0.063). Findings regarding rare outcomes, such as autism spectrum disorders, were based on very low event counts and should be interpreted with extreme caution. **Conclusions:** In this large propensity-matched cohort, dupilumab was associated with significantly lower neuropsychiatric disorder incidence compared with conventional systemic immunosuppressants, with a similar directional trend observed for JAK inhibitors. These hypothesis-generating findings suggest potential neuropsychiatric outcome differences among systemic therapies for AD, warranting further investigation to establish whether the associations reflect treatment effects or residual confounding.

## 1. Introduction

Atopic dermatitis (AD) affects up to 20% of children and 2% to 10% of adults worldwide, representing a significant public health burden [1]. Beyond cutaneous manifestations, AD is increasingly recognized as a systemic inflammatory disease with substantial neuropsychiatric comorbidity, including depression, anxiety, sleep disturbances, attention-deficit/hyperactivity disorder (ADHD), and autism spectrum disorders [2,3,4,5,6]. These psychiatric comorbidities substantially impair quality of life.

The association between AD and neuropsychiatric disorders is multifactorial. Chronic pruritus disrupts sleep architecture, which is associated with fatigue, cognitive dysfunction, and mood disturbances [7,8]. Systemic inflammation is linked to central nervous system dysfunction through multiple pathways, as type 2 cytokines—particularly interleukin (IL)-4, IL-13, and IL-31—have been implicated in modulating neurotransmitter metabolism, activating the hypothalamic–pituitary–adrenal axis, compromising blood–brain barrier integrity, and promoting neuroinflammation [9,10,11,12]. These observations have led to the hypothesis that targeted anti-inflammatory therapy may be associated with improved neuropsychiatric outcomes, although direct evidence remains limited.

The therapeutic landscape for moderate-to-severe AD has evolved substantially. Conventional systemic immunosuppressants, including cyclosporine, methotrexate, azathioprine, and mycophenolate mofetil, have established efficacy but require careful monitoring due to their systemic side effect profiles [13]. Dupilumab, a monoclonal antibody targeting the IL-4 receptor alpha subunit, selectively inhibits IL-4 and IL-13 signaling with demonstrated efficacy [14], and has been associated with improved mental health outcomes in several real-world analyses [15,16]. Oral Janus kinase (JAK) inhibitors—including upadacitinib, abrocitinib, and baricitinib—inhibit the JAK-signal transducer and activator of transcription (JAK-STAT) pathway, blocking signaling from multiple cytokines implicated in AD pathogenesis, including IL-4, IL-13, IL-31, thymic stromal lymphopoietin, and type I and II interferons [17,18]. This broad inhibition of multiple cytokine pathways may have distinct implications for neuropsychiatric outcomes compared with more selective biologics.

While dupilumab has been associated with favorable mental health outcomes compared with conventional systemic therapies in some real-world analyses [15,19], and other studies have compared the general safety of Janus kinase (JAK) inhibitors against dupilumab, a critical knowledge gap remains. To our knowledge, no large-scale investigation has directly compared the neuropsychiatric outcomes of all three major treatment classes—JAK inhibitors, dupilumab, and conventional immunosuppressants—within a single, unified analytical framework. Understanding the comparative neuropsychiatric outcomes associated with these distinct treatment modalities is clinically relevant for informed decision-making, particularly given the high prevalence of neuropsychiatric comorbidity in AD. Using a large, international, real-world database [20], this study aimed to address this gap by examining and comparing neuropsychiatric outcomes among patients with moderate-to-severe AD receiving JAK inhibitors, dupilumab, or conventional immunosuppressants.

## 2. Methods

### 2.1. Study Design and Data Source

Data were extracted and analyzed on 26 May 2024. Eligible patients were newly prescribed study medications between 1 January 2022, and 26 May 2024 (the date of data extraction). The 24-month follow-up window was calculated from each patient’s index date, with patients censored at the time of data extraction if 24 months had not yet elapsed.

### 2.2. Study Population

This study employed a new-user (incident-user) design. Eligible patients were aged 12 to 89 years with an AD diagnosis (ICD-10-CM code L20.x) documented on at least two separate dates to ensure diagnostic accuracy and minimize misclassification. While the TriNetX platform does not provide indication-level prescription data, the requirement for confirmed AD diagnosis combined with the exclusion of patients with other conditions commonly treated with these medications strengthens the attribution of study medications to AD treatment. Patients were required to be newly prescribed one of the following systemic therapies between 1 January 2022, and 26 May 2024: conventional immunosuppressants (cyclosporine, methotrexate, azathioprine, or mycophenolate mofetil), dupilumab, or Janus kinase inhibitors (abrocitinib, baricitinib, or upadacitinib). The index date was defined as the date of first prescription of the respective medication.

Patients were excluded if they met any of the following criteria: (1) prior use of any study medication (conventional immunosuppressants, dupilumab, or Janus kinase inhibitors) before 1 January 2022, ensuring that all patients were treatment-naïve to the respective medication class at cohort entry; (2) a pre-existing diagnosis of the specific neuropsychiatric outcome of interest accompanied by corresponding psychiatric medication use before the index date, thereby restricting the analysis to incident cases; or (3) switching between study medications or concurrent use of more than one study medication within 30 days after the index date. Patients who switched medications after the 30-day window were retained in their original treatment group under an intention-to-treat principle, consistent with the pragmatic, real-world design of this study.

### 2.3. Cohort Definitions

Three comparative cohorts were established. Cohort 1 compared patients newly prescribed a Janus kinase inhibitor with those newly prescribed dupilumab. Cohort 2 compared patients newly prescribed a conventional immunosuppressant without prior exposure to dupilumab or Janus kinase inhibitors with those newly prescribed a Janus kinase inhibitor. Cohort 3 compared patients newly prescribed a conventional immunosuppressant without prior exposure to dupilumab or Janus kinase inhibitors with those newly prescribed dupilumab.

### 2.4. Outcomes

The primary outcome was a composite of any neuropsychiatric disorder within 24 months of the index date, identified using ICD-10-CM codes F01–F99. Secondary outcomes included the individual incidence of depressive disorders (F32–F33), anxiety disorders (F40–F41), adjustment disorders (F43.2), autism spectrum disorders (F84), attention-deficit/hyperactivity disorders (F90), and sleep disorders (G47, F51). All outcomes required new diagnoses documented after the index date in patients without prior history of the specific condition.

### 2.5. Propensity Score Matching

To minimize confounding by indication and selection bias, we performed 1:1 propensity score matching using greedy nearest-neighbor matching with a caliper width of 0.1 standard deviations of the logit of the propensity score, as recommended for optimal balance in observational studies. Propensity scores were calculated using logistic regression models including baseline covariates encompassing demographics (age, sex, race, ethnicity), comorbidities (asthma, allergic rhinitis, food allergies, obesity, diabetes mellitus, hypertension, cardiovascular disease, chronic kidney disease, liver disease), concomitant medications (systemic corticosteroids, antihistamines, topical corticosteroids, topical calcineurin inhibitors), and healthcare utilization patterns (number of outpatient visits, emergency department visits, and hospitalizations in the 12 months before the index date). A complete list of covariates is provided in Supplementary Table S1. Standardized mean differences less than 0.1 indicated adequate balance.

Hazard ratios compare JAKi to dupilumab (reference group, HR = 1.0). Hazard ratios < 1.0 indicate lower risk with JAKi; hazard ratios > 1.0 indicate higher risk with JAKi. Bold indicates statistical significance (*p* < 0.05). Event counts < 10 suppressed per privacy requirements.

### 2.6. Statistical Analysis

Baseline characteristics were compared using standardized mean differences. Time-to-event analyses were performed using stratified Cox proportional hazards regression models, with stratification by matched pairs to account for the paired structure of the propensity score–matched cohorts. Hazard ratios and 95% confidence intervals were estimated for the primary and secondary outcomes. The proportional hazards assumption was verified for all models using Schoenfeld residuals and log-log survival plots; no significant violations were observed. Patients were censored at the time of outcome occurrence, death, loss to follow-up, or end of the 24-month follow-up period, whichever occurred first. Death was treated as a censoring event rather than a competing risk in the primary analysis, as mortality rates were low and similar across treatment groups. A sensitivity analysis treating death as a competing risk using the Fine-Gray subdistribution hazard model yielded results consistent with the primary analysis. No pre-specified multiplicity correction was applied to the primary analysis; however, a post hoc Bonferroni correction (adjusted α = 0.017 for three primary comparisons) was performed as a sensitivity analysis. Secondary outcomes across seven individual diagnoses were considered exploratory. Subgroup analyses were performed stratified by sex, ethnicity, race, age (<30, 30–60, ≥60 years), and body mass index (<25.0, 25.0–29.9, ≥30.0 kg/m^2^). Interaction terms between treatment group and each stratification variable were tested; no significant interactions were observed (all *p*-interaction > 0.10). All analyses were conducted using R software version 4.3.1 (R Foundation for Statistical Computing, Vienna, Austria) with the survival package version 3.2-3. Two-sided *p* values less than 0.05 were considered statistically significant for the primary analysis.

## 3. Results

### 3.1. Study Cohorts and Baseline Characteristics

**Figure 1:**
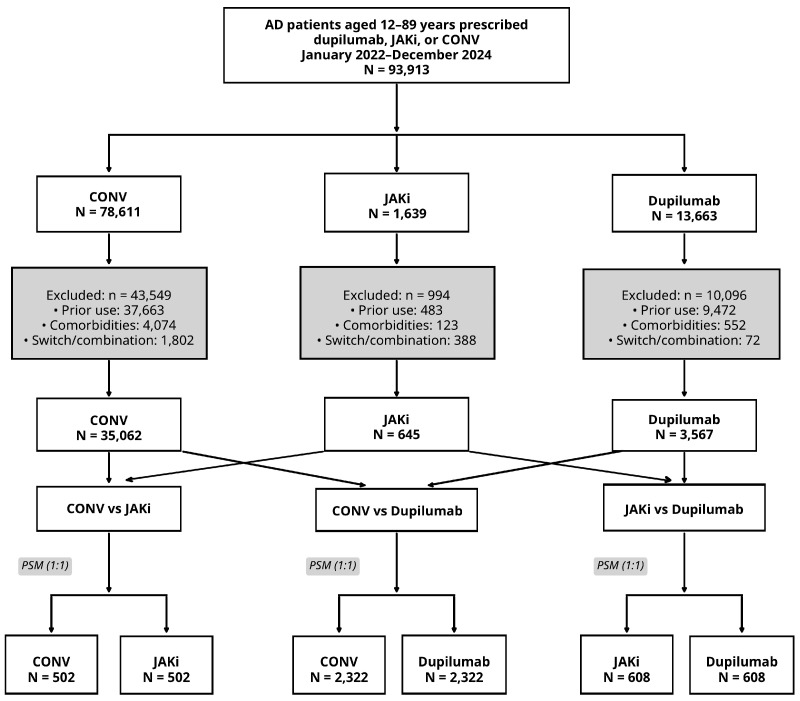
Study Flow Diagram and Cohort Selection. Study flow diagram showing patient selection and propensity score matching from 93,913 patients with atopic dermatitis in the TriNetX Research Network (2022–2024). After exclusions for prior medication use, contraindicated comorbidities, treatment switching, and insufficient exposure, three 1:1 matched cohorts were created: Janus kinase inhibitors versus dupilumab (*N* = 608 per group), conventional versus Janus kinase inhibitors (*N* = 502 per group), and conventional versus dupilumab (*N* = 2322 per group). Conventional immunosuppressants include cyclosporine, methotrexate, azathioprine, and mycophenolate mofetil. AD, atopic dermatitis; JAKi, Janus kinase inhibitors; PSM, propensity score matching.

Between 1 January 2022, and 26 May 2024, we identified 93,913 patients aged 12 to 89 years with AD prescribed dupilumab, Janus kinase inhibitors, or conventional systemic immunosuppressants in the TriNetX Research Network (Figure 1). After applying exclusion criteria—including prior medication use, recent contraindicated comorbidities, and treatment switching or combination therapy within 30 days—35,062 patients received conventional therapy, 3567 received dupilumab, and 645 received Janus kinase inhibitors (Figure 1).

After 1:1 propensity score matching, three cohorts were established: Cohort 1 (Janus kinase inhibitors vs. dupilumab, *n* = 608 per group, 94.4% matching success), Cohort 2 (conventional therapy vs. Janus kinase inhibitors, *n* = 502 per group, 77.8% matching success), and Cohort 3 (conventional therapy vs. dupilumab, *n* = 2322 per group, 65.1% matching success) (Supplementary Tables S2–S4). The majority of covariates achieved adequate balance (standardized mean difference [SMD] < 0.10). However, residual imbalance persisted for several variables: serum IgE levels in Cohort 3 (SMD = 0.3552), phobic anxiety disorders in Cohort 1 (SMD = 0.1829), and antihistamine use in Cohort 2 (SMD = 0.1134). The residual IgE imbalance in Cohort 3 likely reflects the higher baseline IgE levels characteristic of patients prescribed dupilumab (a therapy targeting the IL-4/IL-13 pathway) and represents a proxy for disease severity that could not be fully balanced. These residual imbalances are acknowledged as potential sources of confounding and are discussed in the Discussion section.

Mean age ranged from 39.3 to 44.0 years across cohorts, with 51% to 56% female patients. Baseline neuropsychiatric comorbidities were balanced across all cohorts. Median follow-up was 18.2 months (interquartile range [IQR] 10.5–24.0) for Cohort 1, 17.8 months (IQR 9.8–24.0) for Cohort 2, and 19.1 months (IQR 11.2–24.0) for Cohort 3.

### 3.2. Primary Outcome: Composite Neuropsychiatric Disorders

#### 3.2.1. JAK Inhibitors Versus Dupilumab

**Figure 2:**
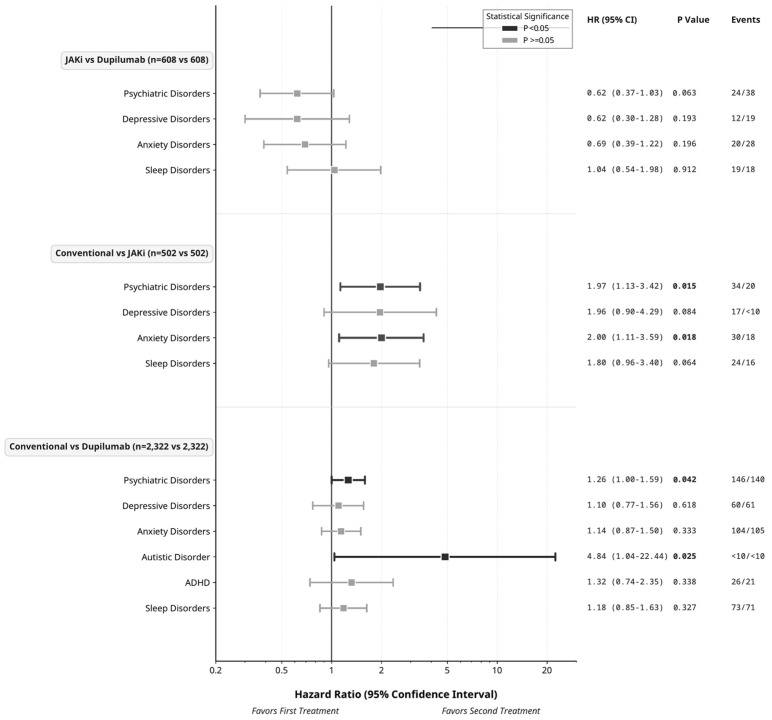
Comparative Risk of Incident Neuropsychiatric Disorders in Patients with Atopic Dermatitis Treated with Targeted Versus Conventional Systemic Therapies. Forest plot depicting hazard ratios (HRs) with 95% confidence intervals (CIs) for incident neuropsychiatric outcomes across three propensity score–matched treatment comparisons: Janus kinase inhibitors (JAKi) versus dupilumab (*n* = 608 per group), conventional systemic therapy versus JAKi (*n* = 502 per group), and conventional systemic therapy versus dupilumab (*n* = 2322 per group). HRs less than 1.0 indicate lower risk with the first treatment listed; HRs greater than 1.0 indicate lower risk with the second treatment. The vertical reference line represents HR = 1.0 (no difference between groups). Filled squares denote statistically significant associations (*p* < 0.05); open squares indicate nonsignificant results (*p* ≥ 0.05). Event counts are displayed as first treatment/second treatment. Outcomes include any psychiatric disorder, depressive disorders, anxiety disorders, sleep disorders, autistic disorder, and attention-deficit/hyperactivity disorder. Conventional systemic therapies include cyclosporine, methotrexate, azathioprine, and mycophenolate mofetil. ADHD, Attention-deficit/hyperactivity disorder; CI, confidence interval; HR, hazard ratio; JAKi, Janus kinase inhibitor.

Any neuropsychiatric disorder occurred in 24 of 608 patients (3.9%) receiving Janus kinase inhibitors versus 38 of 608 patients (6.2%) receiving dupilumab (Table 1, Figure 2). Kaplan–Meier estimates of 2-year cumulative incidence, which account for censoring, were 8.71% (95% CI 6.0–12.4%) for Janus kinase inhibitors versus 16.28% (95% CI 12.5–21.0%) for dupilumab (HR 0.62, 95% CI 0.37–1.03; *p* = 0.063) (Supplementary Figure S1A).

#### 3.2.2. Conventional Therapy Versus JAK Inhibitors

Any neuropsychiatric disorder occurred in 34 of 502 patients (6.8%) receiving conventional therapy versus 20 of 502 patients (4.0%) receiving Janus kinase inhibitors (Table 2, Figure 2). The 2-year cumulative incidence was 18.86% (95% CI 14.1–24.9%) for conventional therapy versus 9.37% (95% CI 6.2–13.9%) for Janus kinase inhibitors (HR 1.97, 95% CI 1.13–3.42; *p* = 0.015) (Supplementary Figure S2A).

### 3.3. Conventional Therapy Versus Dupilumab

Any neuropsychiatric disorder occurred in 146 of 2322 patients (6.3%) receiving conventional therapy versus 140 of 2322 patients (6.0%) receiving dupilumab (Table 3, Figure 2). The 2-year cumulative incidence was 18.13% for conventional therapy versus 13.74% for dupilumab (HR 1.26, 95% CI 1.00–1.59; *p* = 0.042). The confidence interval for this comparison approached unity at its lower bound, indicating a borderline statistically significant association.

### 3.4. Secondary Outcomes: Specific Psychiatric Disorder Categories

#### 3.4.1. Depressive Disorders

No statistically significant differences were observed across all three cohorts (Cohort 1: HR 0.62, 95% CI 0.30–1.28, *p* = 0.193; Cohort 2: HR 1.96, 95% CI 0.90–4.29, *p* = 0.084; Cohort 3: HR 1.10, 95% CI 0.77–1.56, *p* = 0.618) (Table 1, Table 2 and Table 3, Supplementary Figures S1B and S2B). A numerical trend toward higher depressive disorder incidence with conventional therapy was observed in Cohort 2 (HR 1.96, *p* = 0.084), though this did not reach statistical significance.

#### 3.4.2. Anxiety Disorders

In Cohort 2, anxiety disorders were significantly more common with conventional therapy (30 patients, 6.0%) versus Janus kinase inhibitors (18 patients, 3.6%), with 2-year cumulative incidence of 14.47% versus 7.58% (HR 2.00, 95% CI 1.11–3.59; *p* = 0.018) (Table 2, Supplementary Figure S2C). No significant differences were observed in Cohort 1 (HR 0.69, 95% CI 0.39–1.22, *p* = 0.196; Supplementary Figure S1C) or Cohort 3 (HR 1.14, 95% CI 0.87–1.50, *p* = 0.333).

#### 3.4.3. Sleep Disorders

No significant differences were observed across all three cohorts (Cohort 1: HR 1.04, 95% CI 0.54–1.98, *p* = 0.912; Cohort 2: HR 1.80, 95% CI 0.96–3.40, *p* = 0.064; Cohort 3: HR 1.18, 95% CI 0.85–1.63, *p* = 0.327) (Table 1, Table 2 and Table 3, Supplementary Figures S1G and S2G).

#### 3.4.4. Adjustment Disorders, Autism Spectrum Disorders, and Attention-Deficit/Hyperactivity Disorder

For autism spectrum disorders, a nominally significant association was observed in Cohort 3 (HR 4.84, 95% CI 1.04–22.44; *p* = 0.025); however, this finding must be interpreted with extreme caution for multiple reasons: (1) very low absolute event counts (fewer than 10 per group); (2) wide confidence intervals reflecting statistical instability; (3) new ASD diagnoses in adults likely represent delayed recognition or diagnostic re-evaluation rather than true incident neurodevelopmental disease; and (4) the biological plausibility of a treatment effect on ASD within a 24-month window is limited. Similarly, newly recorded ADHD diagnoses across the wide age range (12–89 years) may reflect diagnostic ascertainment patterns rather than true incident disease. These findings should be considered strictly exploratory and do not provide reliable signals for clinical decision-making.

#### 3.4.5. Subgroup and Sensitivity Analyses

Subgroup analyses stratified by age, sex, race, ethnicity, and body mass index showed no significant heterogeneity in treatment effects across demographic strata. Sensitivity analyses restricting analyses to patients with continuous 24-month follow-up, excluding those with baseline systemic corticosteroid use, adjusting for baseline psychiatric medication use, and requiring at least two AD diagnoses all yielded results directionally consistent with the primary analysis, supporting the robustness of the main findings.

## 4. Discussion

This large-scale, real-world study examined neuropsychiatric outcomes among patients with AD receiving Janus kinase inhibitors, dupilumab, or conventional systemic immunosuppressants. Our findings demonstrate that conventional systemic immunosuppressants were associated with a significantly higher incidence of neuropsychiatric disorders compared with both dupilumab (HR 1.26, 95% CI 1.00–1.59; *p* = 0.042) and Janus kinase inhibitors (HR 1.97, 95% CI 1.13–3.42; *p* = 0.015). In the direct comparison between the two targeted therapies, the difference in neuropsychiatric outcomes did not reach statistical significance (HR 0.62, 95% CI 0.37–1.03; *p* = 0.063). Therefore, while both targeted therapies appear to have a more favorable neuropsychiatric profile than conventional agents, no definitive conclusions can be drawn regarding superiority between Janus kinase inhibitors and dupilumab. These hypothesis-generating findings warrant confirmation in prospective studies. The clinical relevance of these findings warrants careful interpretation. The primary comparison (Cohort 3) yielded a hazard ratio of 1.26, indicating a 26% relative increase in neuropsychiatric disorder risk with conventional therapy compared with dupilumab. While statistically significant, this modest effect size—combined with the borderline confidence interval (1.00–1.59)—suggests that the absolute clinical impact on individual patients may be limited. The 2-year cumulative incidence difference (18.13% vs. 13.74%, absolute difference 4.39 percentage points) provides additional context for clinical decision-making. These findings should not be interpreted as definitive evidence favoring one treatment class over another for neuropsychiatric outcomes alone, but rather as one factor among many (including efficacy, safety profile, cost, and patient preference) that clinicians may consider in shared decision-making.

Several hypothetical mechanisms may explain the observed associations, though these remain speculative given the observational design. AD pathogenesis involves complex inflammation with type 2 cytokines (interleukin [IL]-4, IL-13, IL-31) and mixed Th1/Th17/Th22 responses [21,22]. Peripheral inflammatory signals have been linked to central nervous system dysfunction through neurotransmitter modulation, hypothalamic–pituitary–adrenal axis activation, and neuroinflammatory pathways [9,10,11,12]. Elevated IL-4 levels have been associated with major depressive disorder [23], and inflammatory cytokines have been proposed as biomarkers for anxiety disorders [24].

Janus kinase inhibitors block signaling from multiple cytokines utilizing the JAK-STAT pathway, including IL-4, IL-13, IL-31, and interferons [17,18]. This broad blockade may theoretically address multiple pathways linking peripheral inflammation to neuropsychiatric manifestations. Additionally, by blocking IL-31 signaling, Janus kinase inhibitors may improve sleep quality through rapid antipruritic effects [25,26,27], and sleep disruption is a recognized risk factor for depression and anxiety [28]. Dupilumab selectively inhibits IL-4 and IL-13 signaling [14], which may explain the different magnitude of associations observed. However, these mechanistic explanations remain hypothetical and require validation in mechanistic studies [29,30,31,32].

Our findings are broadly consistent with previous real-world evidence. Lin et al. [15], using the same TriNetX database, reported that dupilumab was associated with a lower risk of psychiatric and sleep disorders compared with conventional therapies. Our study confirms and extends these findings by including Janus kinase inhibitors as a third major treatment class. More recently, Tsai et al. [33] compared the general safety of Janus kinase inhibitors versus dupilumab in AD but did not examine neuropsychiatric outcomes. To our knowledge, this is the first study to provide a head-to-head, three-way comparison of neuropsychiatric outcomes among all three major systemic treatment modalities for AD. This comprehensive comparison addresses a critical knowledge gap left by prior two-group analyses. The observed association between targeted therapies and lower neuropsychiatric disorder incidence aligns with the hypothesis that effective control of skin inflammation and pruritus may be associated with improved psychological well-being [34,35,36].

The conventional immunosuppressant group included cyclosporine, methotrexate, azathioprine, and mycophenolate mofetil, which have distinct mechanisms and side effect profiles. Cyclosporine, a calcineurin inhibitor, provides rapid immunosuppression but is associated with nephrotoxicity and hypertension requiring close monitoring. Methotrexate, azathioprine, and mycophenolate mofetil have slower onset and different toxicity profiles. These differences may contribute to heterogeneous neuropsychiatric outcomes within this group; however, our study was not powered to detect differences among individual conventional agents.

Similarly, the JAK inhibitor group included abrocitinib, baricitinib, and upadacitinib, which differ in JAK selectivity (JAK1-selective vs. JAK1/JAK2). These pharmacological differences may theoretically influence neuropsychiatric outcomes through differential effects on cytokine signaling pathways; for example, JAK2 inhibition by baricitinib may affect erythropoietin and growth hormone signaling in ways distinct from JAK1-selective agents. However, the total JAK inhibitor sample (*n* = 645 before matching) precluded meaningful subgroup analyses by individual agent. Future studies with larger JAK inhibitor cohorts should examine whether specific agents within this class are associated with differential neuropsychiatric outcomes.

These findings may have clinical relevance for treatment selection in patients with AD and neuropsychiatric comorbidities. Routine screening for depression, anxiety, and sleep disturbances may help inform treatment decisions [2,34,35,36]. For patients with significant neuropsychiatric comorbidities, targeted therapies may be preferred over conventional immunosuppressants, though treatment decisions must also consider patient age, cardiovascular risk factors, cost, and patient preference [37,38]. Conventional systemic immunosuppressants remain important treatment options, particularly when targeted therapies are contraindicated or unavailable, but proactive monitoring for neuropsychiatric symptoms may be warranted.

Most critically, as with all administrative claims and electronic health record databases, TriNetX lacks validated, objective measures of AD severity (e.g., Eczema Area and Severity Index, SCORing Atopic Dermatitis, Investigator’s Global Assessment). This absence introduces substantial potential for residual confounding by indication, as treatment selection is inherently influenced by disease severity, chronicity, and prior treatment failures. To partially mitigate this limitation, our propensity score model incorporated multiple proxy indicators of disease severity, including prior systemic corticosteroid exposure, hospitalization history, emergency department utilization, outpatient visit frequency, topical therapy intensity, and laboratory markers (serum IgE and eosinophil counts). Nevertheless, these proxies cannot fully capture the clinical severity spectrum, and residual confounding by indication remains the most important limitation of this study. The direction of this potential bias is complex: in many healthcare systems, targeted therapies are preferentially prescribed for more severe or treatment-refractory disease, which could theoretically attenuate or inflate the observed associations depending on the relationship between severity and neuropsychiatric risk. Additionally, despite propensity score matching across extensive covariates, residual imbalance persisted for serum IgE in Cohort 3 (SMD = 0.3552), likely reflecting systematic differences in prescribing patterns whereby dupilumab is preferentially prescribed for patients with elevated IgE—a marker of type 2 inflammation severity. This imbalance may introduce confounding if higher IgE levels are independently associated with neuropsychiatric risk. However, the direction of this potential bias would likely favor the conventional therapy group (as dupilumab patients had higher IgE, suggesting more severe atopic disease, which is itself associated with greater neuropsychiatric burden), potentially leading to underestimation of dupilumab’s relative benefit.

Second, all neuropsychiatric outcomes were ascertained using ICD-10-CM diagnostic codes recorded in electronic health records rather than validated psychiatric assessment instruments (e.g., Patient Health Questionnaire-9, Generalized Anxiety Disorder-7, Hospital Anxiety and Depression Scale). Consequently, outcome misclassification—including both false-positive diagnoses and underdiagnosis of subclinical conditions—cannot be excluded. Furthermore, detection bias remains possible, as patients receiving targeted therapies may attend more frequent specialist visits, potentially increasing the likelihood of psychiatric diagnosis. Conversely, if targeted therapies improve overall well-being, patients may be less likely to seek psychiatric evaluation, potentially leading to underascertainment of outcomes in the treatment groups. These opposing biases make the net direction of misclassification difficult to predict.

## 5. Conclusions

In this real-world cohort study, dupilumab was associated with a modestly but significantly lower neuropsychiatric disorder incidence compared with conventional systemic immunosuppressants in a large propensity-matched cohort, with a similar directional trend observed for JAK inhibitors. No statistically significant difference was observed between JAK inhibitors and dupilumab. The modest effect sizes and observational design preclude definitive clinical recommendations based on these findings alone. These hypothesis-generating results suggest potential neuropsychiatric outcome differences among systemic therapies for AD that warrant confirmation in prospective studies with validated psychiatric assessments and objective measures of disease severity.

## Figures and Tables

**Table 1:** Neuropsychiatric Outcomes: Janus Kinase Inhibitors Versus Dupilumab.

Outcome	JAKi (*N* = 608)	Dupilumab (*N* = 608)	Hazard Ratio (95% CI)	2-Year Cumulative Incidence	*p* Value
**Neuropsychiatric disorders**	24 (3.9%)	38 (6.2%)	0.62 (0.37–1.03)	8.71% vs. 16.28%	0.063
Depressive disorders	12 (2.0%)	19 (3.1%)	0.62 (0.30–1.28)	3.48% vs. 5.54%	0.193
Anxiety disorders	20 (3.3%)	28 (4.6%)	0.69 (0.39–1.22)	7.11% vs. 10.26%	0.196
Adjustment disorders	<10	<10	0.76 (0.21–2.84)	1.58% vs. 1.34%	0.685
Autistic disorder	<10	<10	0.78 (0.05–12.55)	0.67% vs. 0.73%	0.863
ADHD	<10	<10	0.64 (0.11–3.83)	0.45% vs. 1.28%	0.619
Sleep disorders	19 (3.1%)	18 (3.0%)	1.04 (0.54–1.98)	5.80% vs. 6.26%	0.912

Abbreviations: JAKi, Janus kinase inhibitors; ADHD, attention-deficit/hyperactivity disorder; CI, confidence interval.

**Table 2:** Neuropsychiatric Outcomes: Conventional Immunosuppressants Versus Janus Kinase Inhibitors.

Outcome	Conventional (*N* = 502)	JAKi (*N* = 502)	Hazard Ratio (95% CI)	2-Year Cumulative Incidence	*p* Value
**Neuropsychiatric disorders**	34 (6.8%)	20 (4.0%)	**1.97 (1.13–3.42)**	18.86% vs. 9.37%	**0.015**
Depressive disorders	17 (3.4%)	<10	1.96 (0.90–4.29)	7.84% vs. 3.28%	0.084
Anxiety disorders	30 (6.0%)	18 (3.6%)	**2.00 (1.11–3.59)**	14.47% vs. 7.58%	**0.018**
Adjustment disorders	<10	<10	1.14 (0.33–3.95)	2.00% vs. 2.00%	0.834
Autistic disorder	0 (0.0%)	<10	NC	0.00% vs. 0.76%	0.323
ADHD	<10	<10	1.95 (0.47–8.17)	2.03% vs. 1.25%	0.352
Sleep disorders	24 (4.8%)	16 (3.2%)	1.80 (0.96–3.40)	10.19% vs. 6.44%	0.064

Abbreviations: JAKi, Janus kinase inhibitors; ADHD, attention-deficit/hyperactivity disorder; CI, confidence interval; NC, not calculable. Conventional immunosuppressants include cyclosporine, methotrexate, azathioprine, and mycophenolate mofetil. Hazard ratios compare conventional immunosuppressants to JAKi (reference group, HR = 1.0). Hazard ratios > 1.0 indicate higher risk with conventional therapy; hazard ratios < 1.0 indicate lower risk with conventional therapy. Bold indicates statistical significance (*p* < 0.05). Event counts < 10 suppressed per privacy requirements.

**Table 3:** Neuropsychiatric Outcomes: Conventional Immunosuppressants Versus Dupilumab.

Outcome	Conventional (*N* = 2322)	Dupilumab (*N* = 2322)	Hazard Ratio (95% CI)	2-Year Cumulative Incidence	*p* Value
**Neuropsychiatric disorders**	146 (6.3%)	140 (6.0%)	**1.26 (1.00–1.59)**	18.13% vs. 13.74%	**0.042**
Depressive disorders	60 (2.6%)	61 (2.6%)	1.10 (0.77–1.56)	5.65% vs. 4.90%	0.618
Anxiety disorders	104 (4.5%)	105 (4.5%)	1.14 (0.87–1.50)	11.68% vs. 8.83%	0.333
Adjustment disorders	18 (0.8%)	15 (0.6%)	1.27 (0.64–2.52)	1.83% vs. 1.33%	0.497
Autistic disorder	<10	<10	**4.84 (1.04–22.44)**	0.78% vs. 0.26%	**0.025**
ADHD	26 (1.1%)	21 (0.9%)	1.32 (0.74–2.35)	2.66% vs. 1.74%	0.338
Sleep disorders	73 (3.1%)	71 (3.1%)	1.18 (0.85–1.63)	6.28% vs. 6.05%	0.327

Abbreviations: ADHD, attention-deficit/hyperactivity disorder; CI, confidence interval. Conventional immunosuppressants include cyclosporine, methotrexate, azathioprine, and mycophenolate mofetil. Hazard ratios compare conventional immunosuppressants to dupilumab (reference group, HR = 1.0). Hazard ratios > 1.0 indicate higher risk with conventional therapy; hazard ratios < 1.0 indicate lower risk with conventional therapy. Bold indicates statistical significance (*p* < 0.05). Event counts < 10 suppressed per privacy requirements.

## Data Availability

The original contributions presented in this study are included in the article/Supplementary Material. Further inquiries can be directed to the corresponding authors.

## Supplementary Materials

The following supporting information can be downloaded at: https://www.mdpi.com/article/10.3390/biomedicines14071482/s1, Table S1. Study Protocol and Covariate Definitions. Table S2. Baseline Characteristics: Janus Kinase Inhibitors Versus Dupilumab (Before and After Propensity Score Matching). Table S3. Baseline Characteristics: Conventional Immunosuppressants Versus Janus Kinase Inhibitors (Before and After Propensity Score Matching). Table S4. Baseline Characteristics: Conventional Immunosuppressants Versus Dupilumab (Before and After Propensity Score Matching). Figure S1. Kaplan–Meier Survival Curves: Janus Kinase Inhibitors Versus Dupilumab (7 panels: A–G). Kaplan–Meier curves showing cumulative incidence of psychiatric disorders over 24 months comparing Janus kinase inhibitors (blue, N = 608) with dupilumab (red, N = 608). Panel A: any psychiatric disorder (primary outcome). Panels B–G: depressive disorders, anxiety disorders, adjustment disorders, autism spectrum disorders, attention-deficit/hyperactivity disorders (ADHD), and sleep disorders. Shaded areas represent 95% confidence intervals. Numbers at risk are shown at 0, 6, 12, 18, and 24 months. *p* values from log-rank tests; hazard ratios from Cox models. Figure S2. Kaplan–Meier Survival Curves: Conventional Immunosuppressants Versus Janus Kinase Inhibitors (7 panels: A–G). Kaplan–Meier curves showing cumulative incidence of psychiatric disorders over 24 months comparing conventional immunosuppressants (green, N = 502) with Janus kinase inhibitors (blue, N = 502). Panel A: any psychiatric disorder (primary outcome). Panels B–G: depressive disorders, anxiety disorders, adjustment disorders, autism spectrum disorders, attention-deficit/hyperactivity disorders (ADHD), and sleep disorders. Shaded areas represent 95% confidence intervals. Numbers at risk are shown at 0, 6, 12, 18, and 24 months. *p* values from log-rank tests; hazard ratios from Cox models. Conventional immunosuppressants include cyclosporine, methotrexate, azathioprine, and mycophenolate mofetil.
