# Supplementary material for: Comparative Neuropsychiatric Outcomes of JAK Inhibitors, Dupilumab, and Conventional Immunosuppressants in Atopic Dermatitis: A Real-World Cohort Study
Source: Biomedicines. 2026 Jun 30;14(7):1482. doi: 10.3390/biomedicines14071482 (PMC13404433; doi:10.3390/biomedicines14071482)

A. Psychiatric disorder

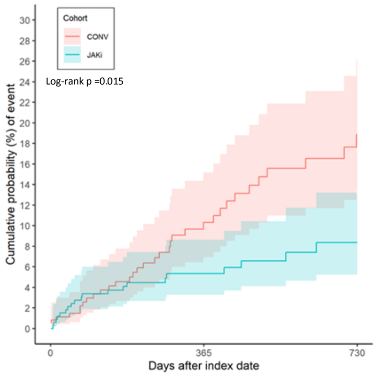

B. Depressive disorder

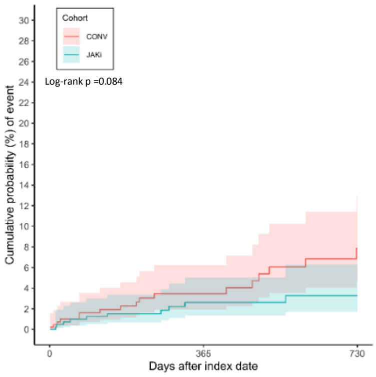

C. Anxiety disorder

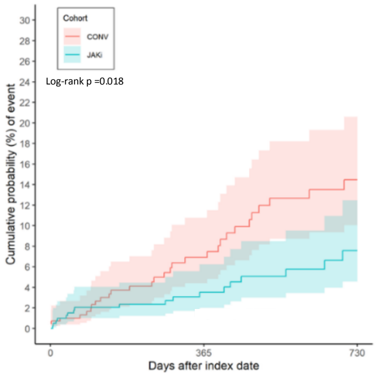

D. Adjustment disorder

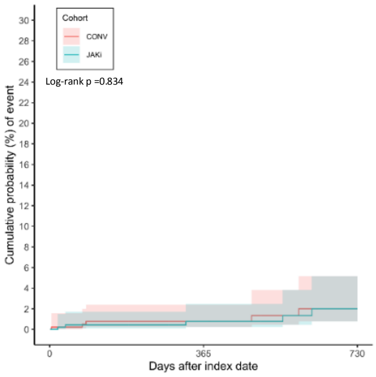

E. Autistic disorder

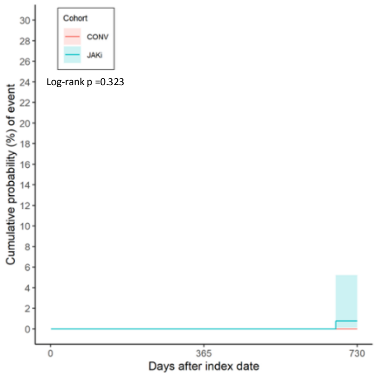

F. ADHD

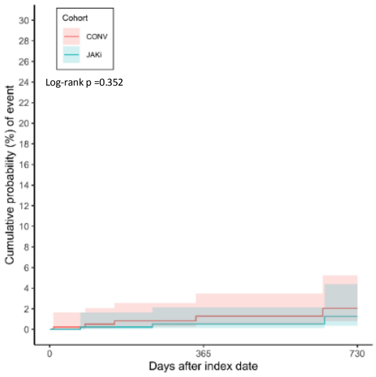

G. Sleep disorder

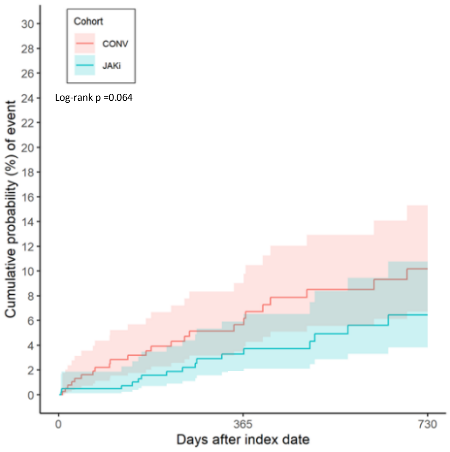

Supplement: Supplementary file 1 [file biomedicines-14-01482-s001.zip › Supplementary Figure S2.pdf]
