# Supplementary material for: Comparative Neuropsychiatric Outcomes of JAK Inhibitors, Dupilumab, and Conventional Immunosuppressants in Atopic Dermatitis: A Real-World Cohort Study
Source: Biomedicines. 2026 Jun 30;14(7):1482. doi: 10.3390/biomedicines14071482 (PMC13404433; doi:10.3390/biomedicines14071482)
Supplement: Supplementary file 1 [file biomedicines-14-01482-s001.zip › Supplementary Tables S2-4.pdf]

Supplementary Table S2. Baseline characteristics: Janus Kinase Inhibitors Versus Dupilumab

	Before PSM			After PSM		
	JAKi	Dupilumab	SMD	JAKi	Dupilumab	SMD
Numbers	644	3564		608	608	
Age at Index(Mean±SD)	39.9 ± 19.4	37.8 ± 20.2	0.1073	39.3 ± 19.4	38.4 ± 19.8	0.0426
Sex, n(%)						
Female	366 (56.8%)	1944 (54.5%)	0.0460	343 (56.4%)	340 (55.9%)	0.0099
Male	266 (41.3%)	1566 (43.9%)	0.0533	254 (41.8%)	256 (42.1%)	0.0067
Ethnicity, n(%)						
Hispanic or Latino	30 (4.7%)	262 (7.4%)	0.1135	30 (4.9%)	30 (4.9%)	0.0000
Not Hispanic or Latino	442 (68.6%)	2353 (66.0%)	0.0557	413 (67.9%)	411 (67.6%)	0.0070
Race, n(%)						
White	266 (41.3%)	1667 (46.8%)	0.1103	251 (41.3%)	273 (44.9%)	0.0731
Black or African American	145 (22.5%)	472 (13.2%)	0.2438	133 (21.9%)	121 (19.9%)	0.0486
Asian	83 (12.9%)	612 (17.2%)	0.1201	81 (13.3%)	62 (10.2%)	0.0971
Nicotine dependence, n(%)	26 (4.0%)	128 (3.6%)	0.0233	21 (3.5%)	21 (3.5%)	0.0000
Tobacco use, n(%)	10 (1.6%)	26 (0.7%)	0.0776	10 (1.6%)	10 (1.6%)	0.0000
Tobacco abuse counseling, n(%)	10 (1.6%)	24 (0.7%)	0.0839	10 (1.6%)	10 (1.6%)	0.0000
Alcohol related disorders, n(%)	10 (1.6%)	24 (0.7%)	0.0839	10 (1.6%)	10 (1.6%)	0.0000
Alcohol abuse counseling and surveillance, n(%)	13 (2.0%)	34 (1.0%)	0.0881	10 (1.6%)	10 (1.6%)	0.0000
History of mental disorder, n(%)						
Mental, Behavioral and Neurodevelopmental disorders, n(%)	68 (10.6%)	275 (7.7%)	0.0988	62 (10.2%)	57 (9.4%)	0.0277
Other anxiety disorders	41 (6.4%)	201 (5.6%)	0.0306	39 (6.4%)	35 (5.8%)	0.0275
Sleep disorders	31 (4.8%)	101 (2.8%)	0.1034	26 (4.3%)	31 (5.1%)	0.0389
Sleep disorders not due to a substance or known physiological condition	10 (1.6%)	12 (0.3%)	0.1260	10 (1.6%)	10 (1.6%)	0.0000
Depressive episode	28 (4.3%)	146 (4.1%)	0.0125	26 (4.3%)	23 (3.8%)	0.0251

Major depressive disorder, recurrent	10 (1.6%)	81 (2.3%)	0.0526	10 (1.6%)	10 (1.6%)	0.0000
Phobic anxiety disorders	10 (1.6%)	10 (0.3%)	0.1338	10 (1.6%)	0 (0.0%)	0.1829
Attention-deficit hyperactivity disorders	12 (1.9%)	24 (0.7%)	0.1065	11 (1.8%)	12 (2.0%)	0.0121
Adjustment disorders	10 (1.6%)	23 (0.6%)	0.0871	10 (1.6%)	10 (1.6%)	0.0000
Asthma	60 (9.3%)	463 (13.0%)	0.1169	59 (9.7%)	55 (9.0%)	0.0226
Vasomotor and allergic rhinitis	49 (7.6%)	398 (11.2%)	0.1222	47 (7.7%)	52 (8.6%)	0.0301
Essential (primary) hypertension	42 (6.5%)	346 (9.7%)	0.1169	41 (6.7%)	46 (7.6%)	0.0319
Co-morbidity, n(%)						
Overweight and obesity	29 (4.5%)	127 (3.6%)	0.0478	27 (4.4%)	25 (4.1%)	0.0163
Hyperlipidemia, unspecified	26 (4.0%)	133 (3.7%)	0.0158	23 (3.8%)	22 (3.6%)	0.0087
Type 2 diabetes mellitus	15 (2.3%)	51 (1.4%)	0.0662	14 (2.3%)	16 (2.6%)	0.0212
Food allergy status	10 (1.6%)	13 (0.4%)	0.1221	10 (1.6%)	10 (1.6%)	0.0000
Pruritus, unspecified	79 (12.3%)	456 (12.8%)	0.0159	75 (12.3%)	74 (12.2%)	0.0050
Noninfective enteritis and colitis	10 (1.6%)	14 (0.4%)	0.1184	10 (1.6%)	10 (1.6%)	0.0000
Cerebrovascular diseases	10 (1.6%)	10 (0.3%)	0.1338	10 (1.6%)	10 (1.6%)	0.0000
Other chronic obstructive pulmonary disease	10 (1.6%)	59 (1.7%)	0.0082	10 (1.6%)	10 (1.6%)	0.0000
Chronic kidney disease (CKD)	10 (1.6%)	21 (0.6%)	0.0937	10 (1.6%)	10 (1.6%)	0.0000
Diseases of liver	10 (1.6%)	90 (2.5%)	0.0688	10 (1.6%)	13 (2.1%)	0.0362
Bullous pemphigoid	17 (2.6%)	10 (0.3%)	0.1976	10 (1.6%)	10 (1.6%)	0.0000
Type 1 diabetes mellitus	12 (1.9%)	10 (0.3%)	0.1542	10 (1.6%)	10 (1.6%)	0.0000
Rheumatoid arthritis, unspecified	10 (1.6%)	10 (0.3%)	0.1338	10 (1.6%)	10 (1.6%)	0.0000
Systemic lupus erythematosus (SLE)	10 (1.6%)	10 (0.3%)	0.1338	10 (1.6%)	10 (1.6%)	0.0000
Dermatopolymyositis	10 (1.6%)	10 (0.3%)	0.1338	10 (1.6%)	10 (1.6%)	0.0000
Sarcoidosis	10 (1.6%)	10 (0.3%)	0.1338	10 (1.6%)	10 (1.6%)	0.0000
Multiple sclerosis	10 (1.6%)	59 (1.7%)	0.0082	10 (1.6%)	10 (1.6%)	0.0000
Transplanted organ and tissue status	0 (0.0%)	10 (0.3%)	0.0750	0 (0.0%)	0 (0.0%)	
Neoplasms	0 (0.0%)	0 (0.0%)		0 (0.0%)	0 (0.0%)	
Myasthenia gravis and other myoneural disorders	0 (0.0%)	0 (0.0%)		0 (0.0%)	0 (0.0%)	

Pemphigus vulgaris	0 (0.0%)	10 (0.3%)	0.0750	0 (0.0%)	0 (0.0%)	
Autoimmune hepatitis	0 (0.0%)	0 (0.0%)		0 (0.0%)	0 (0.0%)	
Amyotrophic lateral sclerosis	0 (0.0%)	0 (0.0%)		0 (0.0%)	0 (0.0%)	
Other medications, n(%)						
Systemic corticosteroids	344 (53.4%)	2190 (61.4%)	0.1630	327 (53.8%)	323 (53.1%)	0.0132
Antihistamines	179 (27.8%)	1012 (28.4%)	0.0134	168 (27.6%)	176 (28.9%)	0.0292
Psycholeptics	103 (16.0%)	712 (20.0%)	0.1039	97 (16.0%)	101 (16.6%)	0.0178
Anxiolytics	88 (13.7%)	472 (13.2%)	0.0123	83 (13.7%)	94 (15.5%)	0.0513
Psychoanaleptics	79 (12.3%)	405 (11.4%)	0.0280	74 (12.2%)	81 (13.3%)	0.0345
Antidepressants	72 (11.2%)	600 (16.8%)	0.1635	67 (11.0%)	72 (11.8%)	0.0258
Hypnotics and sedatives	40 (6.2%)	146 (4.1%)	0.0958	39 (6.4%)	40 (6.6%)	0.0067
azathioprine	10 (1.6%)	14 (0.4%)	0.1184	10 (1.6%)	10 (1.6%)	0.0000
Health care utilization pattern, n(%)						
Visit: Emergency	47 (7.3%)	182 (5.1%)	0.0910	43 (7.1%)	44 (7.2%)	0.0064
Visit: Inpatient Encounter	41 (6.4%)	243 (6.8%)	0.0182	38 (6.3%)	41 (6.7%)	0.0200
Preventive Medicine Services	19 (2.9%)	196 (5.5%)	0.1270	19 (3.1%)	22 (3.6%)	0.0273
Lab exam (Mean±SD)						
Eosinophils/100 leukocytes in Blood	4.49 ± 5.47	5.05 ± 5.14	0.5864	4.56 ± 5.56	4.71 ± 4.76	0.0329
IgE [Units/volume] in Serum, Plasma or Blood	2,181 ± 4,293	1,829 ± 4,743	0.0859	2,379 ± 4,443	1,285 ± 1,319	0.0304

PSM, propensity score matching.

SMD, standardized mean difference. Items with <10 occurrences cannot calculate SMD.

BMI, body mass index.

Supplementary Table S3. Baseline characteristics: Conventional Immunosuppressants Versus Janus Kinase Inhibitors

	Before PSM			After PSM		
	CONV	JAKi	SMD	CONV	JAKi	SMD
Numbers	33938	640		502	502	
Age at Index(Mean±SD)	33.5 ± 18.9	39.9 ± 19.5	0.3349	41.6 ± 20.7	40.2 ± 19.9	0.0650
Sex, n(%)						
Female	19505 (57.5%)	365 (57.0%)	0.0089	257 (51.2%)	279 (55.6%)	0.0879
Male	13664 (40.3%)	263 (41.1%)	0.0169	237 (47.2%)	214 (42.6%)	0.0922
Ethnicity, n(%)						
Hispanic or Latino	3569 (10.5%)	30 (4.7%)	0.2213	22 (4.4%)	27 (5.4%)	0.0462
Not Hispanic or Latino	20464 (60.3%)	440 (68.8%)	0.1773	359 (71.5%)	348 (69.3%)	0.0480
Race, n(%)						
White	15117 (44.5%)	262 (40.9%)	0.0729	197 (39.2%)	208 (41.4%)	0.0447
Black or African American	4158 (12.3%)	145 (22.7%)	0.2767	134 (26.7%)	118 (23.5%)	0.0736
Asian	6142 (18.1%)	83 (13.0%)	0.1420	60 (12.0%)	68 (13.5%)	0.0478
Nicotine dependence, n(%)	1586 (4.7%)	29 (4.5%)	0.0068	22 (4.4%)	26 (5.2%)	0.0374
Tobacco use, n(%)	125 (0.4%)	10 (1.6%)	0.1224	10 (2.0%)	10 (2.0%)	0.0000
Tobacco abuse counseling, n(%)	705 (2.1%)	10 (1.6%)	0.0385	10 (2.0%)	10 (2.0%)	0.0000
Alcohol related disorders, n(%)	811 (2.4%)	10 (1.6%)	0.0595	10 (2.0%)	10 (2.0%)	0.0000
Alcohol abuse counseling and surveillance, n(%)	44 (0.1%)	10 (1.6%)	0.1569	10 (2.0%)	0 (0.0%)	0.2016
History of mental disorder, n(%)						
Mental, Behavioral and Neurodevelopmental disorders, n(%)	3356 (9.9%)	66 (10.3%)	0.0141	60 (12.0%)	58 (11.6%)	0.0124
Other anxiety disorders	3236 (9.5%)	41 (6.4%)	0.1157	37 (7.4%)	39 (7.8%)	0.0151
Sleep disorders	1362 (4.0%)	26 (4.1%)	0.0025	25 (5.0%)	23 (4.6%)	0.0187
Sleep disorders not due to a substance or known physiological condition	1070 (3.2%)	10 (1.6%)	0.1050	10 (2.0%)	10 (2.0%)	0.0000
Depressive episode	2204 (6.5%)	28 (4.4%)	0.0936	25 (5.0%)	24 (4.8%)	0.0092

Major depressive disorder, recurrent	278 (0.8%)	12 (1.9%)	0.0917	15 (3.0%)	10 (2.0%)	0.0640
Phobic anxiety disorders	10 (0.0%)	12 (1.9%)	0.1909	10 (2.0%)	10 (2.0%)	0.0000
Attention-deficit hyperactivity disorders	337 (1.0%)	10 (1.6%)	0.0507	10 (2.0%)	10 (2.0%)	0.0000
Adjustment disorders	292 (0.9%)	10 (1.6%)	0.0642	15 (3.0%)	10 (2.0%)	0.0640
Asthma						
Vasomotor and allergic rhinitis	3003 (8.8%)	48 (7.5%)	0.0492	45 (9.0%)	45 (9.0%)	0.0000
Essential (primary) hypertension	3457 (10.2%)	42 (6.6%)	0.1311	43 (8.6%)	39 (7.8%)	0.0291
Co-morbidity, n(%)	1012 (3.0%)	59 (9.2%)	0.2628	41 (8.2%)	50 (10.0%)	0.0625
Overweight and obesity	6664 (19.6%)	79 (12.3%)	0.2000	71 (14.1%)	71 (14.1%)	0.0000
Hyperlipidemia, unspecified	1369 (4.0%)	30 (4.7%)	0.0320	33 (6.6%)	25 (5.0%)	0.0683
Type 2 diabetes mellitus	1429 (4.2%)	26 (4.1%)	0.0074	24 (4.8%)	22 (4.4%)	0.0191
Food allergy status	52 (0.2%)	17 (2.7%)	0.2139	15 (3.0%)	13 (2.6%)	0.0242
Pruritus, unspecified	345 (1.0%)	13 (2.0%)	0.0829	11 (2.2%)	12 (2.4%)	0.0133
Noninfective enteritis and colitis						
Cerebrovascular diseases	256 (0.8%)	10 (1.6%)	0.0756	10 (2.0%)	10 (2.0%)	0.0000
Other chronic obstructive pulmonary disease	313 (0.9%)	10 (1.6%)	0.0578	10 (2.0%)	10 (2.0%)	0.0000
Chronic kidney disease (CKD)	822 (2.4%)	15 (2.3%)	0.0051	10 (2.0%)	12 (2.4%)	0.0272
Diseases of liver	142 (0.4%)	10 (1.6%)	0.1157	10 (2.0%)	10 (2.0%)	0.0000
Bullous pemphigoid	169 (0.5%)	10 (1.6%)	0.1056	10 (2.0%)	10 (2.0%)	0.0000
Type 1 diabetes mellitus	184 (0.5%)	10 (1.6%)	0.1001	10 (2.0%)	10 (2.0%)	0.0000
Rheumatoid arthritis, unspecified	17 (0.1%)	10 (1.6%)	0.1697	10 (2.0%)	10 (2.0%)	0.0000
Systemic lupus erythematosus (SLE)	11 (0.0%)	10 (1.6%)	0.1727	10 (2.0%)	10 (2.0%)	0.0000
Dermatopolymyositis	35 (0.1%)	0 (0.0%)	0.0454	0 (0.0%)	0 (0.0%)	
Sarcoidosis	170 (0.5%)	10 (1.6%)	0.1052	0 (0.0%)	10 (2.0%)	0.2016
Multiple sclerosis	10 (0.0%)	0 (0.0%)	0.0243	0 (0.0%)	0 (0.0%)	
Transplanted organ and tissue status	0 (0.0%)	0 (0.0%)		0 (0.0%)	0 (0.0%)	
Neoplasms	12 (0.0%)	10 (1.6%)	0.1722	0 (0.0%)	10 (2.0%)	0.2016
Myasthenia gravis and other myoneural disorders	35 (0.1%)	10 (1.6%)	0.1611	0 (0.0%)	10 (2.0%)	0.2016

Pemphigus vulgaris	10 (0.0%)	0 (0.0%)	0.0243	0 (0.0%)	0 (0.0%)	
Autoimmune hepatitis	10 (0.0%)	0 (0.0%)	0.0243	0 (0.0%)	0 (0.0%)	
Amyotrophic lateral sclerosis	10 (0.0%)	0 (0.0%)	0.0243	0 (0.0%)	0 (0.0%)	
Other medications, n(%)						
Systemic corticosteroids	33483 (98.7%)	341 (53.3%)	1.2534	337 (67.1%)	338 (67.3%)	0.0042
Antihistamines	7510 (22.1%)	179 (28.0%)	0.1351	186 (37.1%)	159 (31.7%)	0.1134
Psycholeptics	3361 (9.9%)	103 (16.1%)	0.1849	102 (20.3%)	92 (18.3%)	0.0505
Anxiolytics	2466 (7.3%)	72 (11.3%)	0.1378	72 (14.3%)	65 (12.9%)	0.0406
Psychoanaleptics	4104 (12.1%)	88 (13.8%)	0.0494	63 (12.6%)	77 (15.3%)	0.0806
Antidepressants	3397 (10.0%)	79 (12.3%)	0.0741	57 (11.4%)	69 (13.7%)	0.0722
Hypnotics and sedatives	795 (2.3%)	40 (6.3%)	0.1936	45 (9.0%)	36 (7.2%)	0.0659
azathioprine	391 (1.2%)	10 (1.6%)	0.0355	16 (3.2%)	10 (2.0%)	0.0753
Health care utilization pattern, n(%)						
Visit: Emergency	1112 (3.3%)	47 (7.3%)	0.1821	43 (8.6%)	41 (8.2%)	0.0144
Visit: Inpatient Encounter	3346 (9.9%)	41 (6.4%)	0.1266	39 (7.8%)	38 (7.6%)	0.0075
Preventive Medicine Services	6691 (19.7%)	19 (3.0%)	0.5475	21 (4.2%)	19 (3.8%)	0.0204
Lab exam (Mean±SD)						
Eosinophils/100 leukocytes in Blood	3.39 ± 3.86	4.49 ± 5.48	0.6862	3.96 ± 4.66	4.75 ± 5.84	0.1078
IgE [Units/volume] in Serum, Plasma or Blood	679 ± 2,274	2,181 ± 4,293	0.1317	530 ± 849	2,408 ± 4,581	0.0927

PSM, propensity score matching.

SMD, standardized mean difference. Items with <10 occurrences cannot calculate SMD.

BMI, body mass index.

Supplementary Table S4. Baseline characteristics: Conventional Immunosuppressants Versus Janus Kinase Inhibitors

	Before PSM			After PSM		
	CONV	Dupilumab	SMD	CONV	Dupilumab	SMD
Numbers	34931	3564		2322	2322	
Age at Index(Mean±SD)	33.6 ± 18.9	37.8 ± 20.2	0.2159	37.2 ± 19.7	37 ± 20.2	0.0108
Sex, n(%)						
Female	20068 (57.5%)	1944 (54.5%)	0.0585	1221 (52.6%)	1222 (52.6%)	0.0009
Male	14094 (40.3%)	1566 (43.9%)	0.0728	1073 (46.2%)	1066 (45.9%)	0.0060
Ethnicity, n(%)						
Hispanic or Latino	3608 (10.3%)	262 (7.4%)	0.1050	186 (8.0%)	190 (8.2%)	0.0063
Not Hispanic or Latino	21324 (61.0%)	2353 (66.0%)	0.1035	1546 (66.6%)	1543 (66.5%)	0.0027
Race, n(%)						
White	15765 (45.1%)	1667 (46.8%)	0.0329	998 (43.0%)	1014 (43.7%)	0.0139
Black or African American	6366 (18.2%)	612 (17.2%)	0.0276	453 (19.5%)	446 (19.2%)	0.0076
Asian	4181 (12.0%)	472 (13.2%)	0.0384	346 (14.9%)	329 (14.2%)	0.0208
Nicotine dependence, n(%)	848 (2.4%)	81 (2.3%)	0.0102	62 (2.7%)	64 (2.8%)	0.0053
Tobacco use, n(%)	180 (0.5%)	13 (0.4%)	0.0227	12 (0.5%)	10 (0.4%)	0.0125
Tobacco abuse counseling, n(%)	37 (0.1%)	10 (0.3%)	0.0398	10 (0.4%)	10 (0.4%)	0.0000
Alcohol related disorders, n(%)	267 (0.8%)	24 (0.7%)	0.0108	20 (0.9%)	15 (0.6%)	0.0249
Alcohol abuse counseling and surveillance, n(%)	10 (0.0%)	0 (0.0%)	0.0239	10 (0.4%)	0 (0.0%)	0.0930
History of mental disorder, n(%)						
Mental, Behavioral and Neurodevelopmental disorders, n(%)	1660 (4.8%)	127 (3.6%)	0.0596	79 (3.4%)	88 (3.8%)	0.0208
Other anxiety disorders	6921 (19.8%)	456 (12.8%)	0.1909	316 (13.6%)	321 (13.8%)	0.0063
Sleep disorders	3371 (9.7%)	201 (5.6%)	0.1514	138 (5.9%)	135 (5.8%)	0.0055
Sleep disorders not due to a substance or known physiological condition	1423 (4.1%)	133 (3.7%)	0.0177	98 (4.2%)	96 (4.1%)	0.0043
Depressive episode	326 (0.9%)	14 (0.4%)	0.0666	14 (0.6%)	10 (0.4%)	0.0240

Major depressive disorder, recurrent	1105 (3.2%)	59 (1.7%)	0.0985	46 (2.0%)	46 (2.0%)	0.0000
Phobic anxiety disorders	835 (2.4%)	51 (1.4%)	0.0701	44 (1.9%)	38 (1.6%)	0.0196
Attention-deficit hyperactivity disorders	345 (1.0%)	21 (0.6%)	0.0451	14 (0.6%)	16 (0.7%)	0.0108
Adjustment disorders	144 (0.4%)	10 (0.3%)	0.0224	10 (0.4%)	10 (0.4%)	0.0000
Asthma						
Vasomotor and allergic rhinitis	3091 (8.8%)	398 (11.2%)	0.0773	234 (10.1%)	282 (12.1%)	0.0658
Essential (primary) hypertension	3575 (10.2%)	346 (9.7%)	0.0176	198 (8.5%)	227 (9.8%)	0.0433
Co-morbidity, n(%)	3533 (10.1%)	275 (7.7%)	0.0842	174 (7.5%)	197 (8.5%)	0.0365
Overweight and obesity	2330 (6.7%)	146 (4.1%)	0.1142	105 (4.5%)	106 (4.6%)	0.0021
Hyperlipidemia, unspecified	1518 (4.3%)	128 (3.6%)	0.0386	78 (3.4%)	89 (3.8%)	0.0254
Type 2 diabetes mellitus	1027 (2.9%)	463 (13.0%)	0.3778	326 (14.0%)	330 (14.2%)	0.0049
Food allergy status	1436 (4.1%)	101 (2.8%)	0.0698	62 (2.7%)	71 (3.1%)	0.0232
Pruritus, unspecified	717 (2.1%)	90 (2.5%)	0.0316	39 (1.7%)	57 (2.5%)	0.0545
Noninfective enteritis and colitis	288 (0.8%)	24 (0.7%)	0.0175	19 (0.8%)	18 (0.8%)	0.0048
Cerebrovascular diseases	178 (0.5%)	23 (0.6%)	0.0179	18 (0.8%)	18 (0.8%)	0.0000
Other chronic obstructive pulmonary disease	192 (0.5%)	26 (0.7%)	0.0226	18 (0.8%)	12 (0.5%)	0.0323
Chronic kidney disease (CKD)	364 (1.0%)	34 (1.0%)	0.0089	16 (0.7%)	23 (1.0%)	0.0330
Diseases of liver	302 (0.9%)	24 (0.7%)	0.0219	11 (0.5%)	17 (0.7%)	0.0334
Bullous pemphigoid	11 (0.0%)	59 (1.7%)	0.1783	11 (0.5%)	10 (0.4%)	0.0064
Type 1 diabetes mellitus	128 (0.4%)	12 (0.3%)	0.0050	10 (0.4%)	10 (0.4%)	0.0000
Rheumatoid arthritis, unspecified	54 (0.2%)	10 (0.3%)	0.0270	10 (0.4%)	10 (0.4%)	0.0000
Systemic lupus erythematosus (SLE)	47 (0.1%)	10 (0.3%)	0.0321	10 (0.4%)	10 (0.4%)	0.0000
Dermatopolymyositis	10 (0.0%)	10 (0.3%)	0.0642	10 (0.4%)	10 (0.4%)	0.0000
Sarcoidosis	11 (0.0%)	10 (0.3%)	0.0631	10 (0.4%)	10 (0.4%)	0.0000
Multiple sclerosis	35 (0.1%)	10 (0.3%)	0.0414	10 (0.4%)	10 (0.4%)	0.0000
Transplanted organ and tissue status	19 (0.1%)	10 (0.3%)	0.0553	10 (0.4%)	10 (0.4%)	0.0000
Neoplasms	0 (0.0%)	0 (0.0%)		0 (0.0%)	0 (0.0%)	
Myasthenia gravis and other myoneural	12 (0.0%)	10 (0.3%)	0.0621	0 (0.0%)	10 (0.4%)	0.0930

disorders						
Pemphigus vulgaris	10 (0.0%)	10 (0.3%)	0.0642	0 (0.0%)	0 (0.0%)	
Autoimmune hepatitis	10 (0.0%)	0 (0.0%)	0.0239	0 (0.0%)	0 (0.0%)	
Amyotrophic lateral sclerosis	10 (0.0%)	0 (0.0%)	0.0239	0 (0.0%)	0 (0.0%)	
Other medications, n(%)						
Systemic corticosteroids	34475 (98.7%)	2190 (61.4%)	1.0540	2140 (92.2%)	2131 (91.8%)	0.0143
Antihistamines	7619 (21.8%)	1012 (28.4%)	0.1523	792 (34.1%)	793 (34.2%)	0.0009
Psycholeptics	3514 (10.1%)	405 (11.4%)	0.0422	273 (11.8%)	283 (12.2%)	0.0133
Anxiolytics	3437 (9.8%)	712 (20.0%)	0.2876	564 (24.3%)	558 (24.0%)	0.0060
Psychoanaleptics	2519 (7.2%)	600 (16.8%)	0.2992	490 (21.1%)	477 (20.5%)	0.0138
Antidepressants	4237 (12.1%)	472 (13.2%)	0.0335	315 (13.6%)	325 (14.0%)	0.0125
Hypnotics and sedatives	817 (2.3%)	146 (4.1%)	0.0997	127 (5.5%)	114 (4.9%)	0.0252
azathioprine	391 (1.1%)	14 (0.4%)	0.0839	19 (0.8%)	14 (0.6%)	0.0256
Health care utilization pattern, n(%)						
Visit: Emergency	3418 (9.8%)	243 (6.8%)	0.1077	181 (7.8%)	207 (8.9%)	0.0405
Visit: Inpatient Encounter	1126 (3.2%)	182 (5.1%)	0.0944	152 (6.5%)	143 (6.2%)	0.0159
Preventive Medicine Services	7026 (20.1%)	196 (5.5%)	0.4482	109 (4.7%)	143 (6.2%)	0.0647
Lab exam (Mean±SD)						
Eosinophils/100 leukocytes in Blood	3.38 ± 3.84	5.05 ± 5.14	0.0935	3.86 ± 4.59	5.18 ± 5.31	0.0020
IgE [Units/volume] in Serum, Plasma or Blood	678 ± 2,271	1,829 ± 4,743	0.3097	751 ± 1,152	2,170 ± 5,532	0.3552

PSM, propensity score matching.

SMD, standardized mean difference. Items with <10 occurrences cannot calculate SMD.

BMI, body mass index.
