# Supplementary material for: Comparative Neuropsychiatric Outcomes of JAK Inhibitors, Dupilumab, and Conventional Immunosuppressants in Atopic Dermatitis: A Real-World Cohort Study
Source: Biomedicines. 2026 Jun 30;14(7):1482. doi: 10.3390/biomedicines14071482 (PMC13404433; doi:10.3390/biomedicines14071482)
Supplement: Supplementary file 1 [file biomedicines-14-01482-s001.zip › Supplementary TableS1.pdf]

Supplementary Table S1. Study Protocol and Covariate Definitions

**Category**	**Details**
Inclusion Criteria	
Atopic Dermatitis	ICD-10: L20
Exclusion Criteria	
Prior Medication Use	Use of any conventional immunosuppressant, dupilumab, or JAK inhibitor before the study period (2022).
Recent Comorbidities	Diagnosis of Tuberculosis (TB), Deep Vein Thrombosis (DVT), Pulmonary Embolism (PE), cancer, or liver cirrhosis within 6 months before the index date.
Treatment Switching	Switching between or combination use of study medications within 1 month after the index date.
Covariate Definitions	ICD-10 / RxNorm / ATC Codes
Tuberculosis	A15.0
Deep Vein Thrombosis	I82.40
Pulmonary Embolism	I26.0
Liver Cirrhosis	K74
Nicotine Dependence	F17, Z71.6, Z72.0
Alcohol-Related Disorders	F10, Z71.4
Mental/Behavioral Disorders	F01-F99
Anxiety Disorders	F41
Sleep Disorders	G47, F51
Depressive Disorders	F32, F33
Phobic Anxiety Disorders	F40
ADHD	F90
Adjustment Disorders	F43.2
Hypertension	I10
Diabetes Mellitus	E10, E11
Hyperlipidemia	E78.5
Overweight and Obesity	E66
Cerebrovascular Diseases	I60-I69
Asthma	J45
COPD	J44
Food Allergy	Z91.01
Allergic Rhinitis	J30
Neoplasms	C00-D49
Rheumatoid Arthritis	M06.9
Liver Diseases	K70-K77
Noninfective Enteritis/Colitis	K50-K52
Chronic Kidney Disease	N18

Pruritus	L29.9
Systemic Lupus Erythematosus	M32
Dermatopolymyositis	M33
Sarcoidosis	D86
Myasthenia Gravis	G70
Multiple Sclerosis	G35
Transplanted Organ Status	Z94
Bullous Pemphigoid	L12.0
Pemphigus Vulgaris	L10.0
Autoimmune Hepatitis	K75.4
Amyotrophic Lateral Sclerosis	G12.21
Medications	
Dupilumab	RxNorm: 1876376
Systemic Corticosteroids	ATC: H02
Methotrexate	RxNorm: 6851
Azathioprine	RxNorm: 1256
Cyclosporine	RxNorm: 3008
Upadacitinib	RxNorm: 2196092
Abrocitinib	RxNorm: 2591476
Baricitinib	RxNorm: 2047232
Antihistamines	VA: AH000
Anxiolytics	ATC: N05B
Antidepressants	ATC: N06A; VA: CN600
Hypnotics and Sedatives	ATC: N05C

ADHD, Attention-Deficit Hyperactivity Disorder; ATC, Anatomical Therapeutic Chemical; COPD, Chronic Obstructive Pulmonary Disease; ICD-10, International Classification of Diseases, Tenth Revision; JAK, Janus kinase; RxNorm, a standardized nomenclature for clinical drugs; VA, U.S. Department of Veterans Affairs.
